# The Effect of the General Data Protection Regulation on Medical Research

**DOI:** 10.2196/jmir.7108

**Published:** 2017-02-24

**Authors:** John Mark Michael Rumbold, Barbara Pierscionek

**Affiliations:** ^1^ Faculty of Science, Engineering and Computing Kingston University London Kingston upon Thames United Kingdom; ^2^ School of Science and Technology 50 Shakespeare Street Nottingham Trent University Nottingham NG1 4FQ United Kingdom; ^3^ Faculty of Science, Engineering and Computing Penrhyn Road Kingston University London Kingston upon Thames KT1 2EE United Kingdom

**Keywords:** pseudonymity, anonymity, untraceability, privacy-preserving protocols, informatics, data reporting, data protection, research ethics

## Abstract

**Background:**

The enactment of the General Data Protection Regulation (GDPR) will impact on European data science. Particular concerns relating to consent requirements that would severely restrict medical data research have been raised.

**Objective:**

Our objective is to explain the changes in data protection laws that apply to medical research and to discuss their potential impact.

**Methods:**

Analysis of ethicolegal requirements imposed by the GDPR.

**Results:**

The GDPR makes the classification of pseudonymised data as personal data clearer, although it has not been entirely resolved. Biomedical research on personal data where consent has not been obtained must be of substantial public interest.

**Conclusions:**

The GDPR introduces protections for data subjects that aim for consistency across the EU. The proposed changes will make little impact on biomedical data research.

## Overview

There have been significant developments in European Union (EU) data protection law recently that will have an impact on health care professionals, particularly those engaged in research and audit. The General Data Protection Regulation (GDPR) has replaced the current legislation and comes into full effect in 2018 [1]. The implications for the handling of health care data of the GDPR will be discussed in this paper. Despite the recent referendum vote in the United Kingdom to leave the EU, the GDPR will continue to be relevant to the United Kingdom, whether this is due to cooperation in European projects or because the United Kingdom continues to be a member of the European Economic Area (EEA).

## The Data Protection Directive

Currently the relevant law in the United Kingdom is the Data Protection Act 1998, which is the United Kingdom’s transposition of the Data Protection Directive (DPD). European directives are not directly enforceable, requiring member states to pass legislation to comply with their requirements. There are derogations (legal exemptions) for research, which in the case of the United Kingdom have been criticized for being too broad. The Local and Regional Development Planning Kantor report for the European Commission criticizes the United Kingdom for disregard of the limitations, stating that the Data Protection Act blatantly violates the Directive by adding “medical research” to the list of medical purposes [2]. The DPD requires a “substantial public interest” for member states to add to the derogations for processing of sensitive personal data (Article 8.4).

Differences between EU member states can result in research ethics committees in United Kingdom denying permission for National Health Service (NHS) data to be transferred to other EU countries (the opposite might also be the case in some circumstances) [3]. These differences have also contributed to the passage of the GDPR as part of the Digital Single Market strategy [4].

## The Law as It Will Be From 2018: The General Data Protection Regulation

The text of the GDPR has recently been agreed after a prolonged trilogue between the European Commission, Parliament, and the Council of Ministers [5]. This legislation will replace the national transpositions of the DPD. Regulations are directly enforceable across the EU. The GDPR comes into full effect on May 25, 2018, although member states are permitted minor differences in interpretation (the European Court of Justice is the ultimate arbiter). This legislation has the potential to affect projects using research data banks and Big Data [6,7]. There had been concerns that a clause inserted by the European Parliament requiring specific consent would prevent significant long-term epidemiological research taking place in the future [8], but this was rejected and the agreed text permits broad consent to “certain areas of research when in keeping with recognized ethical standards” (Recital 33) [9]. Broad consent is not blanket or open consent [10] although some commentators argue that blanket or open consent is acceptable for biobank and databank research as the risks are minimal and do not vary for different projects [11]. Another possibility is consent to a form of governance [12]. Open consent without any ongoing regulation or communication about proposed projects would be potentially problematic. Dynamic consent offers advantages for an engaged community of participants but might not be considered beneficial by some individuals [13].

The derogations for research without consent have been expanded to specifically include medical research where “in the public interest” (Recital 51). How public interest will be defined has not been elaborated, but European jurisprudence demands member states satisfy a high threshold where human rights are involved (eg, a “pressing social need” [14]). This standard would not be required for the conduct of medical research using databanks, but it might exclude all commercial research for “me too” drug development (drugs that offer no advantages over drugs already on the market), arrangements that have no evidence of benefit sharing, or simply require that projects address issues of public importance regardless of the profits made [15]. This requirement reflects public attitudes in the United Kingdom to the use of health care data, where there is resistance to use of public data for commercial ventures unless the research could not happen without commercial involvement [16,17].

## Anonymization

Data protection law only applies to personal data—that is, data that does directly or can indirectly identify an individual [18-20]. The simple deletion of name and address is usually insufficient to constitute anonymization (it has been demonstrated that the combination of 3 pieces of data could identify 87% of US residents: 5-digit zip code, birth date, and sex) [21]. The United Kingdom Information Commissioner’s Office currently treats pseudonymized data as anonymous where it is used by a third party who does not possess the requisite key code. Truly anonymized data cannot be linked back to an individual (which means that verification of data is not possible by any means). Pseudonymized data typically has identifiers removed and replaced with a unique key code (there is also 2-way cryptography; 1-way cryptography is considered anonymized). This key code can be used to trace the data back to an individual, enabling any safety concerns to be acted upon and for data to be verified. This is the approach that the United Kingdom Care.data project on the use of NHS electronic health records for data research has been taking [22]. The GDPR will require changes in practice, as it confirms in Recital 26 that pseudonymized data must be treated as personal data (in line with the previous Article 29 Working Party opinion) [18]. That position results from the increased vulnerability of data subjects who could potentially be identified compared to the protection afforded them with true anonymisation—if the key code is hacked, then all the data can be linked to an individual once more.

## Consent

Consent presumed by failure to opt-out, or change preticked boxes, will no longer be permitted (unless covered by the derogations)—consent will need to be by a “clear, affirmative action” (Article 4.11). These changes would have arguably made the abandoned Care.data project [23] illegal, despite the passage of enabling legislation that exempted general practitioners from the common law duty of confidentiality when fulfilling their contractual duties to pass on health care data. Care.data relied on an opt-out for legitimacy [22]. The exercise of this opt-out was not straightforward. The numbers opting out far exceeded the estimates and the capacity for the Health and Social Care Information Centre (now NHS Digital) to process in a timely manner. The problems included omission of those who opted out from calls for NHS screening programs, even though this was not the intention of those exercising this right. NHS Digital currently relies on pseudonymization, which the GDPR states is categorized as a matter of law as personal data. It is not entirely clear whether or not third parties without access to the key code could treat pseudonymized data as anonymized (as is currently the case in the United Kingdom). Key codes are a potential vulnerability due to accidental or malicious disclosure, which is one of the justifications for pseudonymized data being classified as personal data. There are no clear indications that there are no future plans to use NHS patient data for research.

Dame Fiona Caldicott reviewed arrangements because of the widespread concerns related to consent [22], and her report led to the cancellation of the Care.data project [23]. The particular issues that were identified include the lack of information about Care.data that made exercising an opt-out an opaque process, the inadequate mechanisms for opting, and the failure of protection for rights and access to the NHS for those who opt out.

The risk of re-identification in the future is impossible to quantify precisely because it cannot be predicted what information will become public [24]. However, as with biobanks, the risks to individuals are lesser compared with studies of medical interventions [25]. Therefore authorization by research ethics committees is acceptable practice, with the requirement that opt-outs be respected unless there are exceptional circumstances.

Although the GDPR comes into force in mid-2018, researchers need to prepare now for the changes it will bring to long-term epidemiological studies. In particular, the categorization of pseudonymized data as personal will require action in some jurisdictions such as the United Kingdom and Greece [26]. The necessary accommodations will require an investment of resources, but this will hopefully ensure that subjects continue to have trust in the integrity of their health care data and the medical research community [27]. The GDPR may still apply should the United Kingdom cease to become a member state of the EU either because the United Kingdom is a member of the EEA or because the United Kingdom retains these instruments as law at least for the short term [28].

Although audit and research are treated differently in law, the boundaries between the 2 activities are blurred [29]. Audit is directly relevant to the monitoring and improvement of quality of health care; therefore, it is included as a primary use of data—Recitals 52-54 and Article 9.2 (h) and (i) of the GDPR make this clear. Audit and health care management are a primary use of health care data, and research is a secondary use—that is, it is a use different from the originally declared purpose (although it is designated a compatible purpose within the GDPR but only for nonsensitive data). If an audit compares health care systems to discover which is most effective, this can also be categorized as research as the practices are not compared to a gold standard, and there is a hypothesis being generated or even tested by finding associations. The recent furor surrounding the Royal Free Trust project in conjunction with Google DeepMind illustrates the debate over the distinction of audit from research [30-32].

## Data Sharing

Dame Fiona Caldicott affirmed in her 2013 report on information governance that “The duty to share can be as important as the duty to protect patient confidentiality” [33].

Data sharing within the EU should not be obstructed because of differences in data protection law under the principles of the Digital Single Market and Article 1(2) of the Data Protection Directive. Data portability and data sharing is an issue with health care data [34], which the European Patients Smart Open Services (epSOS) project attempted to address [35]. The GDPR addresses data portability under Article 20, stating that the data subject has the right to receive their data in an appropriate format without hindrance and for data to be transferred between data controllers where technically feasible. The Bundestag is currently considering an eHealth bill with the same aim of improving portability of data [36]. This will facilitate the ability of patients to move between health care providers without unnecessary duplication of tests.

## Conclusions

The Digital Single Market aims for improved data sharing across the EU, which will facilitate cross-border health care and research. Harmonization will be improved under the GDPR with a concomitant raising of standards for some countries, although there is still room for national differences according to the reasonable expectations of different publics. This advance makes cross-border projects more easily ethically justifiable and more feasible [37]. The requirements for anonymization have not been changed, except to clarify that pseudonymized data must still be considered as personal data. The GDPR will facilitate medical research, *except* where it is research not considered in the public interest. In that case, more demanding requirements for anonymization will entail either true anonymization or consent. It is likely there will be more projects that require either consent or authorization, since many projects currently use pseudonymization. There is still an unresolved issue over third parties with access to pseudonymized data.

## Abbreviations

DPD: Data Protection Directive
EEA: European Economic Area
epSOS: European Patients Smart Open Services
EU: European Union
GDPR: General Data Protection Regulation
NHS: National Health Service
